# HCV Proteins and Immunoglobulin Variable Gene (IgV) Subfamilies in HCV-Induced Type II Mixed Cryoglobulinemia: A Concurrent Pathogenetic Role

**DOI:** 10.1155/2012/705013

**Published:** 2012-05-29

**Authors:** Giuseppe Sautto, Nicasio Mancini, Laura Solforosi, Roberta A. Diotti, Massimo Clementi, Roberto Burioni

**Affiliations:** Laboratorio di Microbiologia e Virologia, Università Vita-Salute San Raffaele, Via Olgettina, 58, 20132 Milano, Italy

## Abstract

The association between hepatitis C virus (HCV) infection and type II mixed cryoglobulinemia (MCII) is well established, but the role played by distinct HCV proteins and by specific components of the anti-HCV humoral immune response remains to be clearly defined. It is widely accepted that HCV drives the expansion of few B-cell clones expressing a restricted pool of selected immunoglobulin variable (IgV) gene subfamilies frequently endowed with rheumatoid factor (RF) activity. Moreover, the same IgV subfamilies are frequently observed in HCV-transformed malignant B-cell clones occasionally complicating MCII. In this paper, we analyze both the humoral and viral counterparts at the basis of cryoglobulins production in HCV-induced MCII, with particular attention reserved to the single IgV subfamilies most frequently involved.

## 1. Introduction

Mixed cryoglobulinemia (MC), an immune complex (IC)-mediated systemic vasculitis mainly involving the small blood vessels, has been observed in a wide variety of diseases, including malignancies, chronic infections, and systemic autoimmune disorders [1, 2]. In symptomatic MC, the presence of cold-precipitable immunoglobulins (cryoglobulins) is frequently associated with the development of vascular, renal, and neurological lesions [3–5]. The vast majority (50–90%) of patients with symptomatic type II mixed cryoglobulinemia (MCII), characterized by lymphoproliferation and by the deposition of mono/oligoclonal IgM antibodies (Abs) with rheumatoid factor (RF) activity bound to oligo/polyclonal IgG, are infected with hepatitis C virus (HCV) [6]. Consistently, more than 40% of chronically HCV-infected patients present MCII, that in a relevant number of patients (10–60%) will eventually develop in symptomatic cryoglobulinemia [7, 8].

It has been demonstrated that antiviral treatment significantly induces remission in HCV-associated MCII and that this effect is highly correlated with effective suppression of viral replication, supporting a direct role of HCV in the pathogenesis of this lymphoproliferative disorder [9]. Furthermore, MC should not be considered an *in situ* or occult B-cell lymphoma, as evidences indicate that its B-cell clonal expansion does not still display the molecular features of a true neoplastic process [10]. As a matter of fact, in more than 50% of symptomatic patients the clinical course is relatively benign, but 5–10% of patients with cryoglobulinemic vasculitis develop B-cell malignancies, in particular B-cell non-Hodgkin lymphomas (B-NHL), as compared with 0.2–2.6% of the overall HCV-infected population [11–15]. A possible role of chronic immune stimulation associated with persistent infection in the pathogenesis of these malignancies has been hypothesized and further confirmed by the sequence analysis of tumor-related immunoglobulin (Ig) gene rearrangements, evidencing a preferential use of the same Ig heavy and light chain variable regions (VH and VL) genes associated with anti-HCV response and with MCII [16–18].

In this paper, after reviewing the main viral features associated with MCII, we will overview the main IgV gene subfamilies described in patients with HCV-related MCII and will evidence their correlation with the anti-HCV humoral response and with the MCII-related neoplastic complications.

## 2. The Liver as a Lymphoid Organ

**Figure 1:**
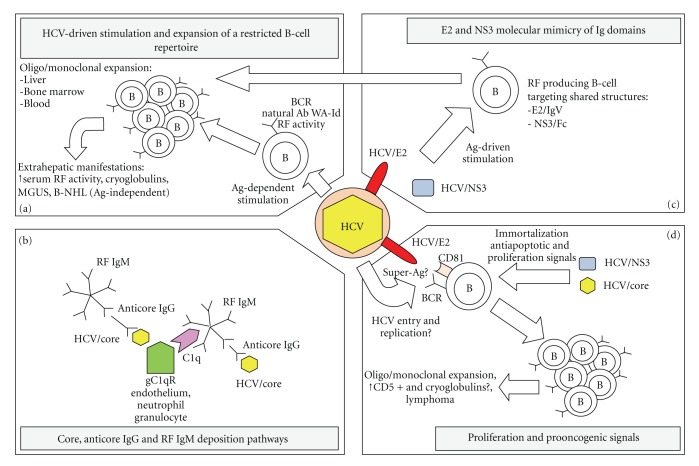
Proposed etiopathogenetic mechanisms involved in the origin of HCV-induced MCII. (a) Direct involvement of HCV infection and of specific HCV Ags in the emergence and maintenance of B-cell expansions, more frequently occurring in the liver and mostly involving RF-producing B cells. This B-cell repertoire is therefore limited and likely coded by few germline genes. These clonal expansions are invariably associated with extrahepatic manifestations, including high serum levels of polyclonal rheumatoid factor activity, cryoglobulins, monoclonal gammopathy of undetermined significance (MGUS), and eventually frank B-cell non-Hodgkin lymphoma (B-NHL). (b) The wide expression of gC1qR on the surface of blood cells, like neutrophil granulocytes, as well as of endothelial cells favors their specific binding to immune complexes containing HCV core protein and may determine their cold precipitation. Alternatively, IgM molecules are good acceptors of C1q, whose binding site is on their Fc portion and, if endowed with RF activity, may precipitate in presence of IgG molecules with specific anticore activity. (c) HCV/E2- and HCV/NS3-induced proliferation and expansion of B-cell clones producing cross-reactive Ig recognizing structures shared between these Ags and discrete Ig regions (i.e., Fc or IgV domains). (d) HCV might initiate a multistage process of lymphomagenesis by replicating in lymphoid cells and expressing proteins that associate with host cell-encoded tumor-suppressing proteins, thereby abrogating their cell-cycle checkpoint functions and predisposing the cell to genetic instability. Alternatively, HCV/E2 binding to CD81, as part of the CD19/CD21/CD81, might provide a strong proliferation signal. Moreover, HCV/E2 could behave as a B-cell super-Ag and directly stimulate proliferation and oligo/monoclonal expansion through its direct binding to BCRs encoded by specific IgV subfamilies.

It is well known that the liver is the main target organ of HCV infection. Within the inflamed liver, particularly in the earliest stages of the disease, there is an accumulation of myeloid and lymphoid cells, including follicular dendritic cells, T and B lymphocytes [19]. Local activation of these cells is thought to play an essential role in perpetuating the chronic inflammatory process and enhancing liver damage [20]. Moreover, intrahepatic B-cell proliferation is often associated with extrahepatic manifestations of HCV infection, including high serum levels of RF activity, cryoglobulins, monoclonal gammopathy of undetermined significance (MGUS), and frank B-NHL, indicating that it has a direct role in HCV-related systemic complications (Figure 1(a)) [21].

Immunohistochemical studies showed that T and B cells frequently accumulate in the portal tracts and organize follicle-like structures (foci), mainly consisting in B cells surrounded by T-cell zones at the periphery. Within these foci there are obvious germinal centres (GCs) where activation, proliferation, differentiation, and maturation of B cells and Ab production may occur similarly to lymphoid organs [22]. This process is rarely observed in patients with other types of hepatitis and seems to contribute to the pathogenesis of HCV immune-related disorders, as suggested by functional and molecular analyses showing that these structures are characterized by B-cell oligo/monoclonal expansion [23]. Indeed, these clonal expansions have been observed in the liver of almost 50% of HCV-infected patients and, less frequently, in their blood and bone marrow [21]. In particular, Sansonno et al. observed a monoclonal pattern only in HCV-infected patients with MC, while Magalini et al. observed it both in MC and non-MC HCV-infected patients, supporting the view that HCV *per se* is able to derange the functions of the immune system [24, 25]. Furthermore, isolated intrahepatic B lymphocytes were shown to produce IgM molecules with RF activity, supporting the intrahepatic origin of HCV-related autoimmune processes [24, 26]. Interestingly, these intrahepatic focus-forming B cells frequently express a restricted repertoire of VH and VL subfamily genes, as discussed more in detail below. Each single focus may derive from a single B cell, with the result that distinct foci contain unrelated B-cell clones with single-antigen (Ag) specificity (Figure 1(a)) [27]. Although continuous viral antigenic stimulation is probably the main factor determining the formation of intrahepatic GCs in HCV-infected patients, the reason why, differently from other hepatotropic viruses, HCV preferentially induces their formation in the liver is currently uncertain. It might be related to the unique virological properties of HCV, including preferential induction of an autoreactive humoral immune response.

Taken together, these observations suggest that, during HCV infection, the liver acts as an important secondary lymphoid organ where autoimmune processes may originate (Table 1). The main viral factors and the specific components of the anti-HCV humoral response involved in this process will be reviewed in the following paragraphs.

## 3. HCV Proteins and B Cells

HCV is an enveloped, positive-stranded RNA virus, characterized by an extreme variability, even within a single host. On the basis of some conserved regions it can be divided in seven genotypes and numerous subtypes [28, 29]. All HCV genotypes have been associated to MCII, although several reports describe its higher prevalence among patients infected with genotypes 1 and 2a [19, 24, 30–34]. However, the differences in the geographical distribution of HCV genotypes may bias this apparent correlation.

HCV genome is approximately 9600 nucleotides long and encodes a polyprotein precursor of about 3000 aminoacids. It is cleaved by viral and host proteases, resulting in a series of structural (core, E1 and E2) and nonstructural proteins (p7, NS2, NS3, NS4A, NS4B, NS5A, and NS5B) [35]. Some HCV proteins have been demonstrated to directly activate important proinflammatory cascades in monocyte and T cells. This activation may serve to lower activation threshold thus enhancing cellular response to Ags, including auto-Ags [36]. This may provide B cells with a pro-inflammatory environment and a myriad of costimulatory signals promoting clonal expansion. Moreover, sequence data on BCR, the presence of HCV Ags in the cryoprecipitates, and a reported correlation between HCV viral load and clinical manifestations of cryoglobulinemia in some patients support the model of an Ag-driven origin for HCV-related lymphoproliferative disorders [30]. In this paragraph we investigate this last point with particular attention to the different HCV proteins possibly involved.

### 3.1. Core Protein

Several reports describe the presence of HCV RNA and HCV proteins in the cryoprecipitate of patients with HCV-related MCII. In particular, the core is supposed to be the most involved viral protein in cryocrit formation, as demonstrated in the skin and renal tissues of HCV-infected patients with MCII-associated active vasculitis and nephropathy, respectively [37]. Nonenveloped core protein is overproduced during virogenesis, and in MCII patients its plasmatic levels have been correlated to cryoglobulinemia-associated symptoms [37, 38]. Indeed, it has been demonstrated that HCV core protein participates in the formation of immune complexes (ICs) and suppresses T-cell response by interacting with the globular domain of C1q complement receptor (gC1qR) (Figure 1(b)) [39, 40]. This interaction may play a key role in determining complement activation, a crucial interdependent regulator of the size and solubility of immune aggregates [41].

Sansonno et al. demonstrated the presence of high levels of HCV core protein in the cryoprecipitates of patients with MCII [42]. These cold-insoluble precipitates included polyclonal IgG and monoclonal IgM molecules with RF activity. In particular, the polyclonal IgG component showed specific reactivity against HCV core protein and, in turn, was linked through its Fc portion to IgM with RF activity. Importantly, cryoprecipitation was directly correlated with anticore IgG concentration in the cryoprecipitate, thus inferring that its production is dependent on their selective binding to the Ag in the presence of IgM molecules with RF activity. Thus, IgM RF acts as an incomplete cryoglobulin, precipitating at low temperature, probably following a conformational change induced by their binding to IgG with anti-core reactivity.

In the same report, the above-described HCV core protein interaction with the gC1qR was proposed as another pathogenetic mechanism, making T cells unable to suppress RF producing B-cell clones generated by chronic antigenic challenge. The presence of gC1qR on the surface of both circulating blood cells and endothelial cells may favor their specific binding to HCV core protein-containing ICs [5, 43]. Moreover, IgM molecules are good acceptors of C1q and indeed can favour indirect binding of HCV core protein to endothelial cell surface (Figure 1(b)) [44].

Finally, HCV core protein has also been shown to promote immortalization in different cell lines, as well as being capable of blocking c-myc induced apoptosis and indeed could have a direct role in the pathogenesis of HCV-related lymphomas [45].

### 3.2. E2 Envelope Glycoprotein

HCV/E2 envelope glycoprotein is another viral protein possibly involved in the development of HCV-related lymphoproliferative disorders [46]. A hint suggesting HCV/E2 role comes from the reported molecular mimicry of its N-terminal hypervariable region (HVR1) with some conserved motifs in selected human Ig variable domains, as well as with the T-cell receptor (TCR) alpha and beta chains (Figure 1(c)) [47]. This molecular mimicry would make anti-HVR1 Abs potentially capable of cross-reacting with other Abs or with TCR. Indeed, the expression of proteins structurally similar to host defense proteins and immunomodulators is an important immune-evasion strategy leading to persistence, already described for other viruses [48–51]. Confirming this, Ferri et al. demonstrated a low pattern of HVR1 mutations in HCV-positive patients with MCII and presenting a monoclonal IgM expansion with RF activity [52]. This low variability in the main target of anti-HCV humoral response was interpreted as a clear sign of impaired response, as already observed in agammaglobulinemia or in otherwise immunosuppressed patients [53, 54]. Interestingly, a direct correlation has been observed between the degree of similarity of HCV/E2 HVR1 to Ig and TCR molecules and the degree of immune escape and persistence in humans and experimentally infected chimpanzees [47]. This indicates that variation in HVR1 sequence is not only correlated with escape from neutralizing Abs but also to an improvement in Ig similarity consistent with a model of immune evasion through mimicry. Overall, this mimicry could determine a chronic stimulation induced also by self-Ags, thus leading to the HCV-related lymphoproliferative disorders.

As a matter of fact, in HCV-associated B-NHL, malignant monoclonal B cells often secrete IgM endowed with RF activity which feature high sequence homology with anti-HCV/E2 Abs [55]. Thus, it has been suggested that these forms of B-NHL may originate from monoclonal proliferation of RF-secreting B cells stimulated as reported above (Figure 1(c)) [52]. Consistently with this observation, Igs cloned from a patient with an HCV-associated B-NHL were shown to bind to HCV/E2 [56].

Finally, it has been postulated that HCV may also exert a direct stimulating activity on B cells following HCV/E2 interaction with the cellular receptor CD81 during the viral particle internalization [57, 58]. In fact, the CD81 tetraspanin on B-cell surface may provide a strong stimulatory signal if activated as part of a complex (CD19/CD21/CD81 complex), together with the activation of BCRs belonging to restricted VH subfamilies and recognizing HCV/E2. Indeed, the ability of E2 to directly engage CD81 in addition to the binding of specific anti-HCV/E2 BCRs may create a powerful stimulatory signal, promoting proliferation (Figure 1(d)). As a possible consequence of this mechanism, CD81 is upregulated in HCV-infected patients, with a further upregulation in patients with MCII. However, conflicting results have been published showing CD81 downregulation, whereas CD19 receptor was upregulated on peripheral B lymphocytes in HCV-infected patients with MCII or B-NHL. In addition, HCV/E2 binding to CD81 induces double-strand DNA breaks and hypermutation, specifically in the VH gene of B cells. This process was demonstrated to be dependent on activation-induced cytidine deaminase and related to an increase in the production of TNF-alpha [59, 60].

### 3.3. NS3 Protein

It is known that NS3 can induce an important humoral and cellular immune response [61–68]. A possible role of NS3 in inducing autoreactive Abs through molecular mimicry has been hypothesized [69]. In particular, IgM reacting against the NS3 helicase domain is present in the cryoprecipitate of all chronically HCV-infected patients with a bone marrow monoclonal B-cell pattern and of a consistent portion (36%) of HCV-related MCII patients [69]. These IgM Abs recognize epitopes located within a region (1238–1279), previously indicated as one of the two immunodominant regions for B cells on NS3 (1250–1334 and 1359–1449). Importantly, these IgM Abs show also reactivity against human IgG and in particular against a unique peptide (Fc345–355) corresponding to the IgG CH3 domain (Figure 1(c)).

On the other hand, the region of NS3 encompassing residues 1406–1415 has been demonstrated to be also a CD8+ T cell epitope [70].

The possible role of HCV/NS3 protein in the pathogenesis of HCV-associated MC is also suggested by another study evidencing NS3 deposits in the kidney of viremic HCV-positive patients with membranoproliferative glomerulonephritis associated with cryoglobulinemia and presenting a mild polyclonal B lymphocytosis [71].

In addition, NS3 has been demonstrated to promote oncogenic transformation and to interact with p53 and interfere with apoptosis, thus contributing singularly or synergistically with the HCV/E2 and core proteins to the development of HCV-related lymphomas [11, 72–74].

## 4. Biased IgV Subfamily Use and Lymphoproliferation

As evidenced above, understanding the nature of the B cell-stimulating Ag resulted to be extremely difficult. One reason relates to the difficulty in isolating expanded cells that are mostly confined to restricted areas of the liver or of the bone marrow. Another reason relates to the complexity of analyzing the BCR specificity of oligoclonal expanded cells, which are present at low concentrations among tissue resident, nonspecific, B-cell populations. A further reason is the difficulty in obtaining reliable information from comparing the sequences of autoreactive BCRs or Abs with those of BCRs or Abs of known specificity.

Some data could be obtained from the cloning of the VH and VL genes into an expression vector and from the testing of their biological activity. As an example, in several studies the use of phage display technology allowed the selection of distinct autoreactive Abs which were further characterized in terms of Ag specificity and biological activity [75]. These studies demonstrated that the VH and VL sequences of the cryoprecipitable monoclonal IgM match those of the IgV genes of monoclonal B cells isolated from patients with MCII [55, 76]. This confirms that in patients with an established monoclonal pattern, the IgM component of ICs represents the circulating counterpart of the BCR expressed on the surface of expanded B cells and, therefore, can be exploited to identify the putative Ag involved in inducing and maintaining B-cell activation.

Interestingly, several other studies demonstrated the recruitment of selected IgV gene subfamilies, the presence of IgV genes mutations compatible with a GC or post-GC derivation, a replacement/silent mutation ratio consistent with the maintenance of a functional structure of the BCR, and the presence of intraclonal heterogeneity evidencing an ongoing Ag-induced maturation [21, 24, 55, 76–80]. Conceivably, the similarity in the structure of the variable BCR region and the restricted recruitment of certain IgV subfamilies, both for heavy and light chains, may account for selection of B cells expressing specific and common reactivity.

In this paragraph we will review the V gene subfamilies most frequently observed in HCV-related lymphoproliferative disorders.

### 4.1. VH1-69

#### 4.1.1. Molecular Characteristics of VH1-69 Subfamily-Derived Abs

Approximately 1.7% of peripheral blood B cells of healthy individuals express the distal VH1-69 gene, as expected for a random use of the total repertoire of functional VH gene regions [81]. Furthermore, this gene segment is expressed in the restricted repertoire of fetal liver B lymphocytes and is thought to be involved in natural immunity [82, 83]. Although minimally used in adult life, this VH subfamily is highly represented in anti-HCV humoral immune response, particularly that directed against the HCV/E2 glycoprotein [84–88].

Interestingly, this subfamily is highly represented also in broadly neutralizing humoral responses directed against other enveloped viruses, such as influenza viruses and HIV [89–92]. In particular, several groups have described the heterosubtypic activity of VH1-69-derived monoclonal antibodies (mAbs) directed against the hemagglutinin (HA) stem region of influenza A viruses [89, 90, 93, 94]. This suggests the presence of a conserved motif in this Ab subfamily determining the observed peculiar features. Indeed, crystallization studies demonstrated that these mAbs interact with the Ag only through the VH1-69-derived heavy chain CDR1 and CDR2 regions, but not with the CDR3 that usually confers Ag specificity to an Ab [95]. In particular, the distinct hydrophobic CDR2 loop encoded by VH1-69 may confer antiviral activity by binding to hydrophobic viral targets [90]. Another peculiar feature of the anti-HA VH1-69-derived mAbs was their extremely long CDR3 region [90]. Interestingly, a very long CDR3 and high levels of somatic mutations were also observed in VH1-69-derived mAbs directed against other viruses, such as HIV [96]. Moreover, similar characteristics were observed also for anti-protein Abs produced in the context of chronic systemic autoimmune diseases [96]. Conversely, for HCV infection, a recent paper reported a shorter CDR3 length of the Ab repertoire in people who spontaneously resolved an acute HCV infection compared to healthy individuals and those with chronically evolving HCV infection, and the authors explain this difference suggesting a mobilization of the Ab repertoire due to clonal selection [76].

#### 4.1.2. VH1-69 and HCV-Related Cryoglobulinemia and Lymphomas

The VH1-69 germline gene is commonly reported in monoclonal IgM observed in HCV-related lymphoproliferative disorders as well as in normal B cells responding to the HCV/E2 viral antigen [52]. Furthermore, analysis of this V region sequence in HCV-infected cryoglobulinemic patients revealed that it undergoes somatic mutation, presumably during affinity maturation. This observation has corroborated the hypothesis that HCV-associated MCII and lymphomas may originate in B cells responding to HCV/E2 glycoprotein, the most involved in stimulation of VH1-69 expressing B cells [79]. Studying patients with HCV-associated type II MC, Carbonari et al. reported that up to 98% of their circulating B cells expressed the VH1-69 gene and that it was frequently associated with the V*κ*3-20 light chain gene [78]. This pairing was also frequently found among B-cell chronic lymphocytic leukemia (B-CLL) clones [105]. Indeed, it has been described that most commonly the association of these two subfamilies forms the WA cross-reactive idiotype (Id) endowed with RF activity [52, 97]. Moreover, it has been observed that the VH1-69 expression is frequently associated with DH3-22 and JH4 rearrangements, especially in patients with HCV-associated MCII [77, 98].

Preferential use of this gene has also been seen in 10–20% of patients with CD5+ B-CLL, and polyclonal activation and expansion of CD5+ B cells occur during interaction between HCV and lymphocytes and are associated with HCV infection and HCV-related MCII [19]. The circulating innate CD5+ cells are in fact believed to be equivalent to murine B-1 cells, which have restricted receptor gene segment usage and are primary source of auto-Abs (IgM). But, in this regard, there are conflicting data, as other groups reported no correlation between the increase of CD5+ B cells and the presence of cryoprecipitate or RF in patients with HCV-related lymphoproliferative disease [77, 80, 99, 100].

Furthermore, it has been observed that the majority of VH1-69-expressing B cells in HCV positive patients had a memory phenotype and express modestly somatically mutated IgM, indicating that a clonal population of memory VH1-69 expressing B cells progressively invades the circulating B-cell compartment of patients with HCV-associated MCII [99]. It has also been reported that the peripheral B-cell repertoire of HCV patients may be represented almost completely by VH1-69 monoclonal B cells [78, 106]. Moreover, some of these clones have CDR3 sequences identical to RF IgMs isolated from patients with MALT neoplasms, with MCII-associated splenic lymphoma, and with leukaemia-like B-cell monoclonal expansion [78, 106]. Thus, originally nonneoplastic VH1-69 B cells responding to Ag stimulation could evade the homeostatic mechanisms that regulate the Ag-driven clonal expansion, and subsequent genetic events may cause further escape from control and lead to absolute lymphocytosis.

Finally, recent evidences suggest that somatic hypermutation, as well as class switching, may significantly alter the germline-determined original BCR reactivity [107, 108]. This may explain why VH1-69-derived IgG Abs are often endowed with a broadly neutralizing anti-HCV activity, whilst their IgM counterpart may feature autoreactive activity. In this regard, Racanelli et al. identified the pauciclonality of the peripheral memory B-cell population as a distinguishing feature of patients who spontaneously resolved an acute HCV infection compared to those chronically evolving HCV infection. This finding, also observed in patients with preneoplastic HCV-associated lymphoproliferative disorders, suggests that the B-cell clones potentially involved in clearance of the virus may also be originally more prone to feature autoimmune characteristics and more susceptible to undergo abnormal expansion [76].

### 4.2. Other VH Subfamilies

B cell mono/oligoclonal expansion of clones expressing VH subfamily gene segments other than VH1-69, such as VH3-7, VH3-21, VH3-23, VH3-30, VH4-34, VH3-48, and VH4-59, seems to be implicated in HCV-related lymphoproliferative disorders [75, 77, 100]. Moreover, as in the case of VH1-69, monoclonal expansion of B-cell clones expressing most of these VH subfamilies is a common feature of a wide variety of autoimmune disorders and lymphomas [75, 100, 101, 108].

#### 4.2.1. VH3-21 and VH3-23

Our group previously reported that the biased VH1-69 gene use in anti-HCV/E2 response may selectively expand B-cell clones reacting against this specific VH subfamily. In particular, it has been observed that the immune repertoire of a patient with HCV-associated MCII contains IgM clones able to react specifically against anti-HCV/E2 Abs belonging to VH1-69 subfamily derived from the same patient. Indeed, we found that 61% of IgMs reactive to anti-HCV/E2 VH1-69-Fab fragments belonged only to two VH subfamilies, VH3-23 (39%) and VH3-21 (22%), that are frequently described in autoimmune disorders [75, 101].

Furthermore, the mutational pattern of selected anti-HCV/E2 IgMs showed that almost all clones featured a high homology to the germline. More in details, differently from VH3-21 subfamily, VH3-23 clones did not feature polyreactivity but showed a binding bias toward the VH1-69-derived IgG1 Fabs. These data suggest that VH3-23 IgM may be naturally prone to recognize specific VH regions within the VH1-69 subfamilies [75, 102].

Overall, the HCV/E2-driven stimulation of the immune system may cause the expansion of specific B cells expressing VH1-69-derived Abs recognized by some natural IgM Ab subfamilies. This could lead to the formation of circulating ICs, and the cross-linking of BCR by auto-Abs may allow a chronic activation and a clonal expansion of anti-HCV/E2 B cells.

#### 4.2.2. VH4-34

The VH4-34 gene is used in about 5% of healthy adult B lymphocytes and is frequently found in diffuse large-cell lymphoma, primary central nervous system lymphoma, B-CLL, and several autoimmune disorders [79].

Furthermore, it is well known that, independently from the associated DH and JH gene segments, as well as from the subfamily and isotype of the paired light chain, the VH4-34 is a naturally autoreactive subfamily. Interestingly, the VH4-34 gene is found in virtually all cases of cold agglutinin disease, where the red blood cell I/I Ags bind to the FR1 domain of selected Ig subfamilies, including VH4-34, with a minor involvement of the CDR3 region [109]. Indeed, the restricted usage of VH genes and the binding outside the CDRs are characteristics of B-cell super-Ags that are supposed to directly activate B cells [110]. As previously mentioned, this could be the case of the HCV/E2 glycoprotein, due to its ability to stimulate the expansion of a restricted set of VH and VL subfamily expressing B cells. Moreover, as previously described, it directly provides a strong proliferation signal to B cells through its interaction with CD81 as part of the BCR complex. A similar behavior has also been described for staphylococcal enterotoxins A and D, that function as human B super-Ags rescuing B cell-expressing VH3 and VH4 (including VH4-34) genes inducing cell survival in *in vitro* experiments, and has been suggested also for gp120 of HIV [96, 111]. Moreover, certain portions of the FRs seem to be important for super-Ag binding, and these would be preserved in a super-Ag selective pressure [109].

Therefore, the high frequency of the VH4-34 gene usage and the intrinsic molecular features of its FR and CDR domains suggest a possible role of yet unknown B-cell super-Ag in driving HCV-related lymphoproliferative disorders [79].

### 4.3. VL Subfamilies

In the majority of clonal B-cell expansions following HCV infection there is a major involvement of V*κ*-expressing B cells, as demonstrated by the highly skewed *κ*/*λ* ratio and as corroborated by the usage of the V*κ* genes belonging to restricted subfamilies, as V*κ*3-15 and V*κ*3-20 [80]. As previously mentioned, the V*κ*3-20 germline gene expression during HCV humoral immune response is frequently associated with the VH1-69 expression in the context of HCV-associated MCII and lymphomas [77, 80, 103].

Giving this restricted V*κ* usage in HCV-positive subjects with a related MCII as well as in those evolving in a B-NHL, some reports suggest an immune attack targeted on idiotypic determinants, as a possible passive or active immunotherapy for HCV-related autoimmune diseases and B-cell lymphomas [104]. In particular, De Re et al. after immunization of an experimental model with a VH4-59/V*κ*3-20 scFv mAb, cloned from a patient with a HCV-related MCII and B-NHL, demonstrated the possible induction of anti-Id Abs directed against conserved V*κ* epitopes. This finding opens to possibilities of a potential therapeutic application of Abs reactive with shared Id for patients with HCV-associated B-cell lymphoproliferative diseases, obviating the need to produce an anti-Id Ab or to use a different Id vaccine for each patient [103]. Consistently, this approach has been applied in some types of B-cell tumors and some autoimmune diseases [112].

## 5. Conclusions

Several viruses are involved in the development of systemic autoimmune-related damage. Over the last few years several studies have firmly demonstrated the close interplay between a primarily hepatotropic virus, such as HCV, and B cells and the role of this interaction in the occurrence of HCV-related autoimmune preneoplastic lymphoproliferative disorders, as MCII. The HCV-induced stimulation of distinct specific B-cell clones expressing specific BCRs, derived from a limited numbers of V gene subfamilies, clearly underlines the role of specific HCV Ags in the initiation of this pathogenetic mechanism. However, there are still many dark areas left, such as the molecular factors determining the breaking of “self tolerance” and those leading from the clonal expansion of a limited number of HCV-specific clones to the neoplastic immortalization of some of them.

Overall, these mechanisms may be seen as escape strategies put forth by HCV to evade the immune response in the course of a persistent infection [47, 113]. This point of view, as well as the approaches followed in the study of HCV-related autoimmune disorders, may be important also in the evaluation of other autoimmune diseases not yet related with an infectious etiology or associated to a given pathogen. The study of the systemic and local B-cell repertoire in the course of diseases, as systemic sclerosis, multiple sclerosis, or specific clinical forms of atherosclerosis, and its comparison with the specific V gene repertoire induced by a given pathogen may be a new way for investigating their real causes opening new doors on the comprehension of their pathogenesis [114–119].

## Figures and Tables

**Table 1:** Schematic representation of viral and humoral factors implicated in the instauration of HCV-related MCII and B-NHL.

	Implicated factors		Associated mechanisms	References
HCV proteins	Core		(i) Complement activation (ii) Cryoprecipitation (iii) ↓ T cell response	[37, 39, 41–43, 45]
			
	E2		(i) Molecular mimicry of IgV domain (ii) Induction of RF (iii) Proliferation and transformation signals following interaction with CD81/BCR (iv) Super-Ag?	[46, 47, 52–59]
			
	NS3		(i) Molecular mimicry of IgG-Fc domain (ii) Oncogenesis	[69, 71]

IgV subfamilies		VH1-69	(i) Expansion of CD5+ B cells? (ii) WA with RF activity (iii) Expansion of natural IgM expressing B cells	[52, 55, 76, 78, 79, 97–99]
			
	Heavy chains	VH4-34	Naturally autoreactive	[79]
		VH4-59, VH3-7, VH3-21, VH3-23, VH3-48 and VH3-30	Mono/oligoclonal expansion following HCV infection	[75, 77, 100–102]
	Light chains	V*κ*3-15		[80]
		V*κ*3-20	WA with RF activity	[77, 80, 97, 103, 104]
